# Characterization of South American Snails of the Genus* Biomphalaria* (Basommatophora: Planorbidae) and* Schistosoma mansoni* (Platyhelminthes: Trematoda) in Molluscs by PCR-RFLP

**DOI:** 10.1155/2016/1045391

**Published:** 2016-11-17

**Authors:** Roberta Lima Caldeira, Tatiana Maria Teodoro, Liana Konovaloff Jannotti-Passos, Pollanah M. Lira-Moreira, Christiane De Oliveira Goveia, Omar dos Santos Carvalho

**Affiliations:** ^1^Grupo de Pesquisa em Helmintologia e Malacologia Médica, Centro de Pesquisas René Rachou, Fiocruz, Avenida Augusto de Lima 1715, 30190-001 Belo Horizonte, MG, Brazil; ^2^Biociências e Biotecnologia em Saúde, Departamento de Entomologia, Centro de Pesquisas Aggeu Magalhães (CPqAm), Recife, PE, Brazil; ^3^Moluscário “Lobato Paraense” Centro de Pesquisas René Rachou, Fiocruz, Avenida Augusto de Lima 1715, 30190-001 Belo Horizonte, MG, Brazil; ^4^Prefeitura Municipal de Belo Horizonte, Av. Afonso Pena 1212, 30130-003 Belo Horizonte, MG, Brazil; ^5^Sessão de Parasitologia, Laboratório de Parasitoses Intestinais e Malacologia, Instituto Evandro Chagas, Rodovia BR-316 Km 7 s/n, Levilândia, 67030-000 Ananindeua, PA, Brazil

## Abstract

The identification of snails of the genus* Biomphalaria* can be done using morphological characteristics which depends on the size of the snails and skill and knowledge of researcher. These methods sometimes are not adequate for identification of species. The PCR-RFLP, using the ITS region of the rDNA, has been used to identify Brazilian species of the genus* Biomphalaria*. Nevertheless, there is a lack of information about snails from other Latin American countries. In addition, some snails may be infected by* Schistosoma mansoni* and when submitted to PCR-RFLP they show molecular profiles different from those previously standardized for the other mollusc species. In this work the molecular profiles of 15 species and the subspecies were established by PCR-RFLP of ITS-rDNA with the enzyme* Dde*I. Moreover, the molecular profiles of host species,* B. glabrata*,* B. straminea*,* B. tenagophila*, and* B. prona*, infected by* S. mansoni* were also established. The molluscs were dissected to permit morphological identification. These results contribute to a correct identification of snails of the genus* Biomphalaria* and detection of these snails infected by* S. mansoni*.

## 1. Introduction

Despite therapeutic advances in the last decade, Schistosomiasis remains one of the most prevalent parasitic diseases worldwide and endemic in 76 countries and territories [1]. In Africa and Neotropical Region there are species of the genus* Biomphalaria* (Gastropoda: Planorbidae) which are intermediate hosts of* Schistosoma mansoni* Sambon, 1907. In Latin America 24 species and one subspecies were registered (Table 1), four of them can be found naturally infected by* S. mansoni*, whereas six were found to be susceptible in the laboratory.

The classical identification of snails of the genus* Biomphalaria* is based on morphological characteristics of the shell and the reproductive system [2]. However, this approach is complicated in cases of inadequate fixation or interspecific similarity. The Polymerase Chain Reaction and Restriction Fragment Length Polymorphism (PCR-RFLP), directed to the internal transcribed spacer (ITS) region of the rDNA gene, has been used with success to resolve these cases. The molecular profile of Brazilian species of the genus* Biomphalaria* using this method has been established [3]. Thus, the specific profile of all these species together will be useful to facilitate interspecific identification in the genus* Biomphalaria*. Besides, the specific identification could be done by comparing the sequences between closely related species [4–6], as well as using the morphology associated with the species-specific PCR [7, 8].

Furthermore, the snails which were collected in the field may be infected with* S. mansoni* during the prepatent period, and when they are submitted to the molecular identification, their DNA is simultaneously amplified with the DNA from the parasite. In this case the molecular profile differs from the profile established for the snail alone.

The aim of the present work is to present the previously species-specific profiles established by PCR-RFLP of ITS-rDNA with* Dde*I and to establish the profiles for* B. glabrata*,* B. tenagophila*,* B. straminea,* and* B. prona* infected by* S. mansoni*.

## 2. Material and Methods

### 2.1. Samples

Of the 24 species registered for Latin America, the Medical Malacological Collection (Fiocruz-CMM) has fifteen species and a subspecies:* B. glabrata*,* B. tenagophila*,* B. occidentalis*,* B. schrammi*,* B. oligoza*,* B. peregrina*,* B. intermedia*,* B. straminea*,* B. kuhniana*,* B. amazonica*,* B. cousini*,* B. prona*,* B. edisoni*,* B. havanensis*,* B. orbignyi*, and* B. tenagophila guaibensis*. The molluscs were dissected to permit morphological identification. DNA of specimens of the Fiocruz-CMM collection was cryopreserved.

*Biomphalaria glabrata*,* B. tenagophila*, and* B. straminea* molluscs and AL, SJ, and LE strains of* S. mansoni* used in this study were maintained and raised in the “Lobato Paraense” Mollusc Rearing of René Rachou Research Center, CPqRR/Fiocruz, in Belo Horizonte, MG, Brazil. The LE strain was isolated, in 1968, from a patient residing in Belo Horizonte, MG (Brazil). The SJ strain was isolated, in 1975, from naturally infected snails from São José dos Campos, São Paulo (Brazil). The AL strain was isolated in 1980 from* B. glabrata* that originated from Alagoas state (Brazil). To obtain specimens of* B. glabrata*,* B. tenagophila*, and* B. straminea* shedding* S. mansoni* cercariae, experimental infection with LE, SJ, and AL strains, respectively, was carried out [9]. However, there was no population of* B. prona* in the “Lobato Paraense” Mollusc Rearing, so the DNA of the snails and the parasite (LE strain) was mixed and amplified together to obtain the profile of this infected species. DNA of adult worms of* S. mansoni* was used for control of amplification.

*Cercaria macrogranulosa, Cercaria caratinguensis*, and* Cercaria ocellifera* were obtained from field snails* Biomphalaria*.

### 2.2. Molecular Techniques

#### 2.2.1. DNA Extraction and PCR-RFLP Assay

Total DNA from* B. glabrata*,* B. tenagophila*, and* B. straminea* infected by* S. mansoni*,* B. prona*, adult worms of* S. mansoni* and* C. macrogranulosa*,* C. caratinguensis*, and* C. ocellifera* were extracted using Wizard Genomic Purification Kit (Promega, Madison, USA) with some modifications. The DNA of all samples was used as template in the PCR-RFLP assay. The entire ITS was amplified using the primers ETTS2 (5′ TAACAAGGTTTCCGTAGGTGAA 3′) and ETTS1 (5′ TGCTTAAGTTCAGCGGGT 3′) anchored, respectively, in the conserved extremities of the 18S and 28S ribosomal genes [10]. The PCR amplification was undertaken in a volume of 10 *μ*L consisting of 1–10 ng template DNA, 10 mM Tris-HCl, pH 8.5, 200 *μ*M of each DNTP, 1.5 mM MgCl_2_, 0.5 U of Taq DNA polymerase, and 50 mM KCl, together with 1.0 pmol of each primer. The reactions were covered with a drop of mineral oil and subjected to the following thermal cycling program: initial denaturation step for 3 min at 95°C, and then 32 cycles with annealing at 54°C for 1 min, extension at 72°C for 2 min, denaturation at 95°C for 45 sec, and a final extension step at 72°C for 5 min. A negative control (no template DNA) was included in all experiments. Three microliters of the amplification products were visualized on silver stained 6% polyacrylamide gels to check the quality of amplification. The remaining 7 *μ*L was mixed with water, and* Dde*I (10–12 units) enzyme was added, together with 1.0 *μ*L of the respective enzyme buffer. The digestion was performed for 3.5 h at 37°C and at 80°C for 20 min for enzyme denaturation and the digestion products were evaluated on silver stained 6% polyacrylamide gels [3].

## 3. Results and Discussion

**Figure 1:**
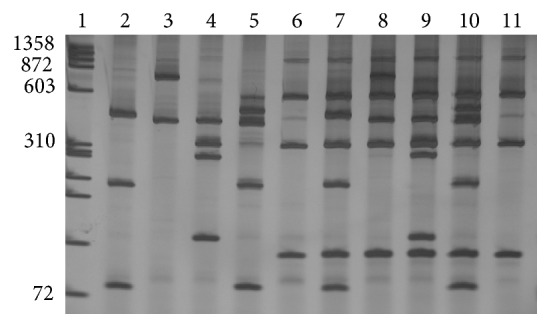
6% silver stained polyacrylamide gel showing restriction profiles obtained by digestion of the ITS region of DNA ribosomal with* Dde*I. Lane 1: molecular size markers Phi X 174/*Hae*III; Lane 2:* Biomphalaria glabrata*; Lane 3:* Biomphalaria tenagophila*; Lane 4:* Biomphalaria straminea*; Lane 5:* Biomphalaria prona*; Lane 6: adult worm of* Schistosoma mansoni*; Lane 7:* B. glabrata* infected by* S. mansoni*; Lane 8:* B. tenagophila* infected by* S. mansoni*; Lane 9:* B. straminea* infected by* S. mansoni*; Lane 10: DNA of* B. prona* with DNA of* S. mansoni*; Lane 11: adult worm of* S. mansoni*. The numbers on the left of the gel represent the value in base pairs (bp).

The PCR amplification resulted in a product of approximately 1200 pb for* Biomphalaria*, one of 800 pb for* S. mansoni*, and both fragments for infected molluscs (data not shown). The RFLP profiles obtained by digesting rDNA ITS with* Dde*I in Figure 1 allow the following: (1) to identify noninfected* B. glabrata*,* B. tenagophila, B. straminea*, and* B. prona*, by observation of species-specific fragments (Lanes 2, 3, 4, and 5); (2) to establish the species-specific profile of* S. mansoni* (Lanes 6 and 11); and (3) to detect by the presence of overlapping species-specific fragments the infection by* S. mansoni* in* B. glabrata*,* B. tenagophila*,* B. straminea*, and* B. prona* (Lanes 7, 8, 9, and 10).

**Figure 2:**
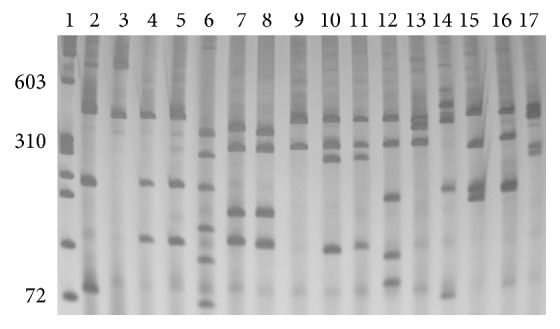
6% silver stained polyacrylamide gel showing restriction profiles obtained by digestion of the ITS region of DNA ribosomal with* Dde*I. Lane 1: molecular size markers Phi X 174/*Hae*III; Lane 2:* Biomphalaria glabrata*; Lane 3:* Biomphalaria tenagophila*; Lane 4:* Biomphalaria tenagophila guaibensis*; Lane 5:* Biomphalaria occidentalis*; Lane 6:* Biomphalaria schrammi*; Lane 7:* Biomphalaria oligoza*; Lane 8:* Biomphalaria peregrina*; Lane 9:* Biomphalaria intermedia*; Lane 10:* Biomphalaria straminea*; Lane 11:* Biomphalaria kuhniana*; Lane 12:* Biomphalaria amazonica*; Lane 13:* Biomphalaria cousini*; Lane 14:* Biomphalaria prona*; Lane 15:* Biomphalaria edisoni*; Lane 16:* Biomphalaria havanensis*; Lane 17:* Biomphalaria orbignyi*. The numbers on the left of the gel represent the value in base pairs (bp).

All 15 species and the subspecies of* Biomphalaria* were dissected and their identification confirmed by analysis of specific diagnostic characters established for each species. In association with morphological identification, the profile of PCR-RFLP was established for these species and is shown in Figure 2.

Studies that incorporate morphological and molecular techniques in taxonomic analysis can generate data that allow a better interpretation and understanding of the biological diversity of the organisms under study. In fact, both the molecular and morphological taxonomy, if properly applied, successfully achieve the same goal [11]. In previous studies, the diagnosis of* S. mansoni* in molluscs has been performed using the LS-PCR [12], the conventional PCR assays for amplification of the Sm1–7 repeated sequence [13], and Loop-Mediated Isothermal Amplification [14] and otherwise the most frequent technique used to the identification of* Biomphalaria* is the PCR-RFLP.

This study has demonstrated the usefulness of the PCR-RFLP technique in the diagnosis of infection by* S. mansoni* in molluscs concurrently with identification of the four intermediate hosts,* B. glabrata*,* B. tenagophila*,* B. straminea*, and* B. prona* (Figure 1). In addition it was possible to identify a unique profile for the cercariae of* S. mansoni*,* C. macrogranulosa*,* C. caratinguensis*, and* C. ocellifera*, obtained from snails* Biomphalaria* collected in the field, after amplification of the ITS region of the rDNA digestion individually with the enzymes* Dde*I,* Alu*I,* Hae*III,* Rsa*I, and* Hpa*II (data not published).

Thus, this molecular biology technique has great utility for generating new knowledge about the taxonomy of molluscs of the genus* Biomphalaria*. Further, from the genetic analysis of various species of* Schistosoma* and* Biomphalaria*, it was observed that intraspecific genetic polymorphism of the parasite is limited while in the mollusc, it is very pronounced, showing the higher relevance of molluscan genetics over parasite genetics in determining the epidemiology of the disease [15]. For example, in adult* B. glabrata*, resistance to* S. mansoni* has been shown to be a dominant single-gene trait that is inherited by Mendelian genetics. In contrast, in juveniles, the genetics of resistance has been shown to involve 5 to 6 genes each with multiple alleles [16]. Additionally, Ittiprasert and Knight report reversing the resistance phenotype of resistant BS-90* B. glabrata* by applying stress in the form of a mild heat pulse before they were exposed to* S. mansoni*, rendering these snails susceptible [17].

## Figures and Tables

**Table 1:** Molluscs of the genus *Biomphalaria* present in Latin America.

Species	Geographical distribution	^a^Susceptibility to *Schistosoma mansoni*
*Biomphalaria amazonica*, Paraense 1966	Brazil, Bolivia, Colombia	EI

*Biomphalaria andecola* (Orbigny, 1835)	Bolivia, Peru, Chile	NI

*Biomphalaria cousini* Paraense, 1966	Brazil, Ecuador	EI

*Biomphalaria edisoni* (Estrada et al., 2006)	Colombia	NI

*Biomphalaria equatoria* (Cousin, 1887)	Ecuador	NI

*Biomphalaria glabrata* (Say, 1818)	Antigua, Brazil, Curacao, Dominica, Guadeloupe, French Guiana, Haiti, Saint Kitts and Nevis, Martinique, Montserrat, Puerto Rico, Dominican Republic, Saint Lucia, Suriname, Venezuela	S

*Biomphalaria havanensis* (Pfeiffer, 1839)	Haiti, Mexico, Puerto Rico, Cuba, Venezuela	EI

*Biomphalaria helophila* (Orbigny, 1835)	Peru, Cuba, Costa Rica, Guatemala, Belize, Haiti, Mexico, Saint Thomas, El Salvador, Dominican Republic, Puerto Rico, Barbados, Nicaragua	EI

*Biomphalaria intermedia* (Paraense & Deslandes, 1962)	Brazil, Argentine	NS

*Biomphalaria kuhniana* (Clessin, 1883)	Suriname, Brazil, Venezuela, Panama, Colombia	NS

*Biomphalaria nicaraguana* (Morelet, 1851)	Nicaragua	NI

*Biomphalaria occidentalis* Paraense, 1981	Brazil, Paraguay, Argentine	NS

*Biomphalaria oligoza* Paraense, 1974	Bolivia, Brazil, Argentine	EI

*Biomphalaria orbignyi* Paraense, 1975	Argentine, Uruguay	EI

*Biomphalaria obstructa* (Morelet, 1849)	Mexico, Puerto Rico, Guatemala, El Salvador, Belize, Cuba	NS

*Biomphalaria pallida* (Adams, 1846)	Jamaica, Cuba	NI

*Biomphalaria peregrina* (Orbigny, 1835)	Ecuador, Bolivia, Chile, Brazil, Paraguay, Peru, Uruguay, Argentine, Colombia	EI

*Biomphalaria prona* (Martens, 1873)	Venezuela	S

*Biomphalaria schrammi* (Crosse, 1864)	French Guiana, Guadeloupe, Brazil	NS

*Biomphalaria sericea* (Dunker, 1848)	Ecuador	EI

*Biomphalaria straminea* (Dunker, 1848)	Venezuela, Suriname, French Guiana, Guyana, Peru, Brazil, Paraguay, Argentine, Dominica, Grenada, Guadeloupe, Martinique, Dominican Republic, Trinidad, Uruguay, Costa Rica	S

*Biomphalaria subprona* (Martens, 1899)	Mexico, Guatemala	NI

*Biomphalaria tenagophila* (Orbigny, 1835)	Argentine, Paraguay, Uruguay, Brazil, Peru, Bolivia	S

*Biomphalaria tenagophila guaibensis* Paraense, 1984	Brazil, Uruguay, Paraguay, Argentine	NS

*Biomphalaria trigyra* (Philippi, 1869)	Peru, Ecuador	NS

a: susceptible = S; not susceptible = NS; experimental infection = EI; not information = NI.
